# Humidified Warmed CO_2_ Treatment Therapy Strategies Can Save Lives With Mitigation and Suppression of SARS-CoV-2 Infection: An Evidence Review

**DOI:** 10.3389/fmed.2020.594295

**Published:** 2020-12-11

**Authors:** Alaa M. M. El-Betany, Enas M. Behiry, Mark Gumbleton, Keith G. Harding

**Affiliations:** ^1^School of Pharmacy and Pharmaceutical Sciences, Cardiff University, Cardiff, United Kingdom; ^2^School of Medicine, Institute of Infection and Immunity, Cardiff University, Cardiff, United Kingdom; ^3^Wound Healing Research Unit, Welsh Wound Innovation Centre, School of Medicine, Cardiff University, Cardiff, United Kingdom

**Keywords:** anti-COVID-19, antiviral, anti-cytokine storm, improve COVID-19 symptoms, carrier gas composition, enhancer antiviral, protect and improve organs function, suppression COVID-19 pandemic

## Abstract

The coronavirus disease (COVID-19) outbreak has presented enormous challenges for healthcare, societal, and economic systems worldwide. There is an urgent global need for a universal vaccine to cover all SARS-CoV-2 mutant strains to stop the current COVID-19 pandemic and the threat of an inevitable second wave of coronavirus. Carbon dioxide is safe and superior antimicrobial, which suggests it should be effective against coronaviruses and mutants thereof. Depending on the therapeutic regime, CO_2_ could also ameliorate other COVID-19 symptoms as it has also been reported to have antioxidant, anti-inflammation, anti-cytokine effects, and to stimulate the human immune system. Moreover, CO_2_ has beneficial effects on respiratory physiology, cardiovascular health, and human nervous systems. This article reviews the rationale of early treatment by inhaling safe doses of warmed humidified CO_2_ gas, either alone or as a carrier gas to deliver other inhaled drugs may help save lives by suppressing SARS-CoV-2 infections and excessive inflammatory responses. We suggest testing this somewhat counter-intuitive, but low tech and safe intervention for its suitability as a preventive measure and treatment against COVID-19. Overall, development and evaluation of this therapy now may provide a safe and economical tool for use not only during the current pandemic but also for any future outbreaks of respiratory diseases and related conditions.

**Graphical Abstract:**
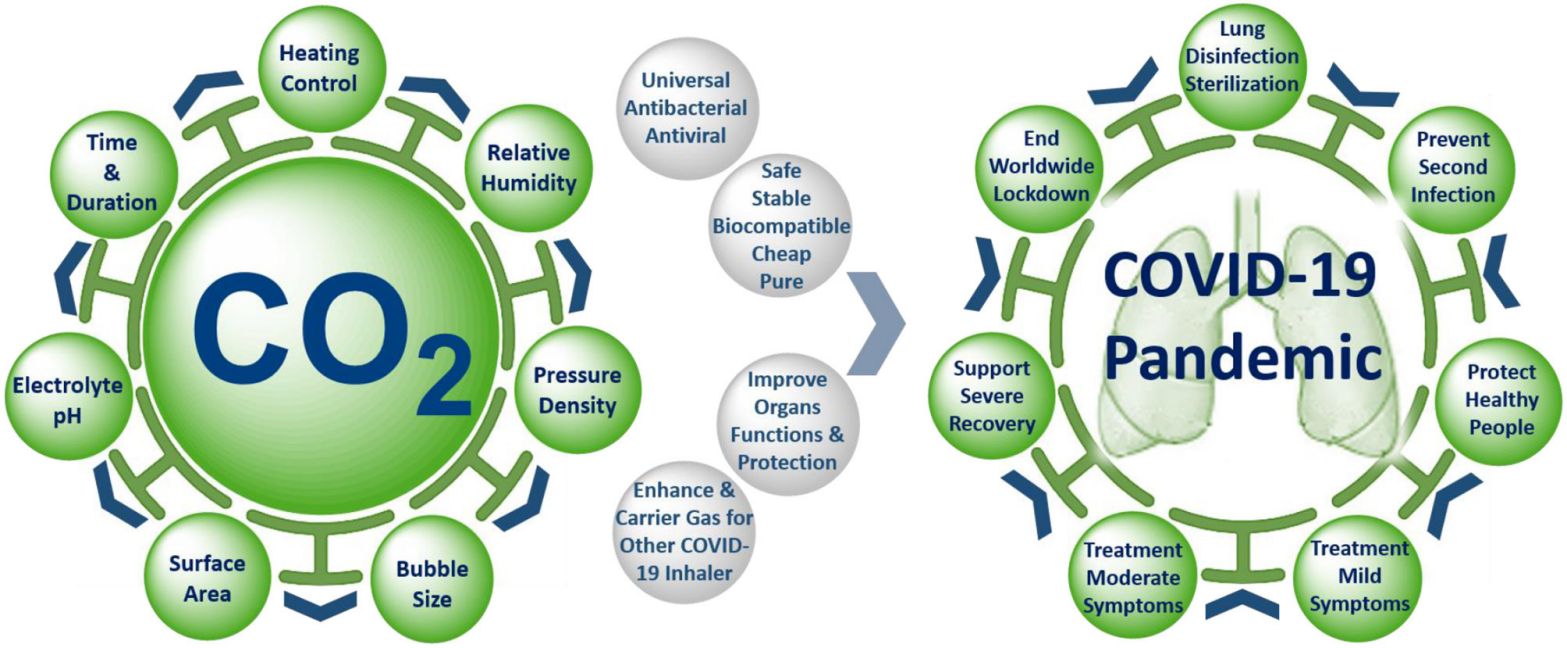
Precise control of the unique properties and intervention parameters of warmed humidified CO_2_ gas make it a promising anti-COVID-19 therapy for mitigation and suppression of SARS-CoV-2 infection.

## Background

The coronavirus disease (COVID-19) outbreak has presented enormous challenges for healthcare systems worldwide and caused terrible societal and economic impacts. There is also an urgent need to address health inequality in treating the current COVID-19 pandemic. Even now, scientists are racing to unravel sometimes conflicting information to understand the source, diagnose, and find effective treatments for SARS-CoV-2, and to conduct clinical trials of antiviral drugs and vaccines. Other COVID-19 mysteries include the appearance of new symptoms, the relation of silent hypoxia and sudden deaths, spikes insignificant vessel blockages, and increased risks of clotting (1). The virus is now known to be able to target a wide variety of cells throughout the human body through ACE2 and TMPRSS2 receptors (2) and is believed to have caused a spike in a rare syndrome: “multi-system inflammatory state requiring intensive care” in children. Furthermore, the mode of transmission and the extent of environmental contamination is yet unknown. While the virus may not technically be airborne, it is definitely borne in the air as aerosols (3).

One of the most critical unanswered questions is why some COVID-19 patients develop severe disease, while others do not? Does the answer hidden in the origin and continuing evolution of SARS-CoV-2 virus mutation into mild and wild different strains (4)? Alternatively, does the answer depend on the two phases of the individual human body immune responses; a protective phase and a damaging phase due to inflammation-cytokine storms (5)? Other questions include whether bacterial co-infections such as bacterial pneumonia and sepsis with antibiotic resistance lead to increased COVID-19 disease severity and mortality (6) and how long it will take to create an effective vaccine. Potential SARS-CoV-2 vaccines have a variety of approaches that depend on viral life cycles (7), and it is estimated that a vaccine will either arrive in 1 or 2 years or will never arrive at all. Even if the vaccine trials are successful, will the new vaccine cover all SARS-CoV-2 mutant strains, and give full immunity to everyone with no issues when translation to clinical practice? Can we produce enough, how much will it cost and who will pay (a particularly important issue in developing countries)? Can the new vaccine stop the threat of a second inevitable wave of coronavirus, or other pandemic viruses emerging to produce a similar situation in the future?

Gas therapy is a highly effective viral inactivation strategy. Carbon monoxide (CO) gas is very flammable and highly poisonous and referred to as the “Silent Killer,” because it binds to the parts of human blood that carry oxygen molecules, so it chemically blocks the body and organs from getting the needed oxygen. However, CO gas has also been shown to have antimicrobial and antiviral activities against infected cells (8), and two clinical trials (NCT02425579, NCT03799874) have demonstrated that the administration of low concentrations of CO is well-tolerated and safe in patients with sepsis-induced ARDS (9, 10). Similarly, while high concentrations of inhaled ozone (O_3_) can damage the lungs, cause chest pain, coughing, shortness of breath, throat irritation, and worsen chronic respiratory diseases such as asthma as well as compromise the ability of the body to fight respiratory infections (11), ozone gas therapy has been demonstrated to inactivate airborne viruses (12) and could inactivate the SARS-CoV-2 virus through oxidizing the sulfhydryl groups in cysteine of the virus-cell (13). There are also at least four ongoing clinical trials (NCT04290871 - NCT04306393 - NCT04305457 - NCT04290858) testing the use of inhaled nitric oxide (NO) gas for patients with COVID-19 (14), as increasing airway NO levels via gas inhalation or precursor molecules may improve oxygenation in COVID-19 subjects (15). As with the other gases, there is another side to NO, which can be harmful due to the formation of highly toxic and irritating nitrogen dioxide (NO_2_) gas and methemoglobinemia (16).

## The Hypothesis and Evidence

**Figure 1:**
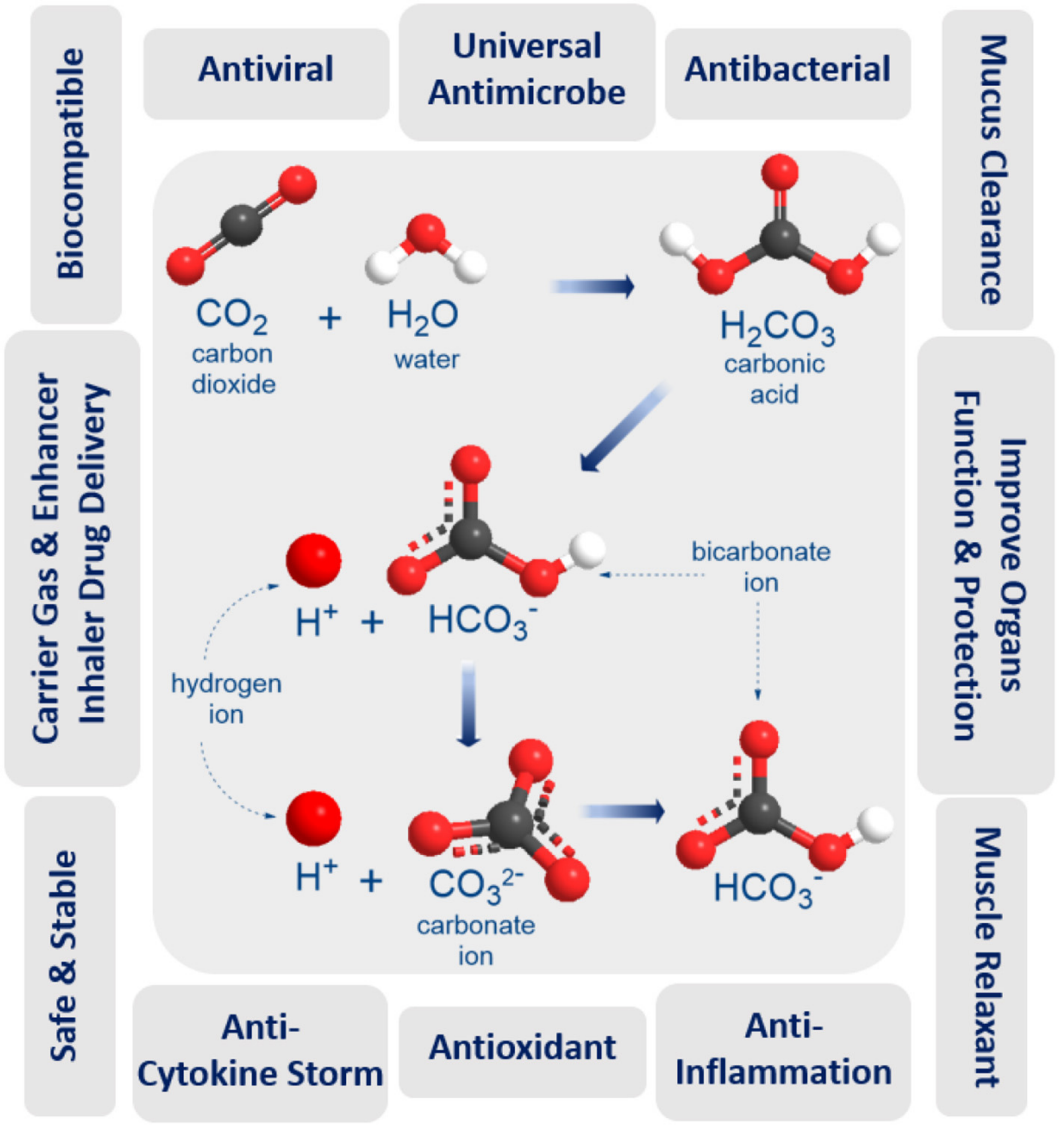
Exceptional physical, biological, and medical properties of warmed humidified CO_2_ gas.

Carbon dioxide (CO_2_) is a fundamental biological gas and has been used for medical purposes for over a century due to its unique properties (Figure 1). Carbon dioxide gas is natural, biocompatible, chemically stable, and safer than any other medical gases (NO, O_3_, or CO). It has been shown to possess antioxidant and anti-inflammatory properties, to improve blood oxygenation and enhance oxygen delivery to organs, to protect and improve lung function, to function as a carrier, or enhancer gas for drug delivery by rapid and direct open airway inhalation with easy administration in home, GP, emergency unit, and ICU settings. These unique biological, physical, and medical properties of CO_2_ make it a promising anti-COVID-19 therapy for mitigation and suppression of SARS-CoV-2 infection. Our hypothesis depends on inhaling precise doses of humidified and warmed CO_2_ medical gas, either alone or as a composite carrier gas with other COVID-19 inhaler medications (bronchodilators, antivirals, antibiotics, or anti-cytokine agents), to disinfect the SARS-CoV-2 virus inside the infected human lung, as a preventative measure to stop coronavirus infection spreading, and to improve the treatment of mild, moderate, and severe COVID-19 symptoms. The following benefits and evidence of using medical carbon dioxide gas support the hypothesis.

### Universal Virucidal and Antimicrobial Activity

Direct inactivation technologies have several limitations against the current virus. Moist, warm CO_2_ gas could become a competitive disinfection technology. Carbon dioxide gas is an antiviral, antibacterial, and anti-infection agent effective not only on solid surfaces but also in aqueous solutions and water treatment settings (17). Heated, un-pressurized carbon dioxide bubbled through wastewater or aqueous media effectively destroys both waterborne bacteria and viruses (18). Moreover, supercritical CO_2_ can in-activate and eliminate coronaviruses from an animal, human tissues and solid surfaces (19–21). Supercritical CO_2_ offers a novel, user-friendly process to sterilize acellular tissue, such as lung matrices, for use in tissue and organ engineering (22). CO_2_ can also enhance the effect of some other antibacterial agents, further improving the protection imparted (17). When breathing is impaired, CO_2_-levels in the human body drop, which creates a favorable environment for bacterial growth and a higher risk of infection. Pure CO_2_ significantly decreased the growth rate of most viruses and bacteria at body temperature; this inhibitory effect of CO_2_ increased exponentially with time (23). This phenomenon could be attributed to unravels the secret of structure and function of the Endothelial Surface Layer (ESL) (24–27). As the venous ESL is probably comprised of nanobubbles of CO_2_, generated from tissue metabolism, that presumably kills the viruses and bacteria exiting to the blood flow on the way to leaving via the lungs (27). Even though the mechanism of inactivation of microorganisms by CO_2_ is not yet resolved, there are a number of hypotheses that have been proposed to explain the unique disinfection action of CO_2_ gas (28).

CO_2_ gas is far superior to other similar gases, with much higher viral inactivation rates at lower temperatures (18–100°C) without the need for pressurization (18, 29). CO_2_ interacts with water moisture to generate carbonic acid (pH 4.18), a reduced pH could affect virus and microbial cell inactivation, as lipid membrane stability is disrupted and permeability to carbon dioxide increases (30, 31). However, a reduction in the pH of the medium is not sufficient to account for the antimicrobial action of CO_2_, since it shows a specific inhibitory effect which is greater than that of the other acids used to lower the pH of media (hydrochloric acid, phosphoric acid, etc.) (32). These acids do not penetrate the microbial cells as easily as carbon dioxide (33). Cheng et al. believe that CO_2_ molecules could enter virus capsids much more easily than H^+^ and inactivate the virus (34). CO_2_-protein binding could also damage the capsid, inactivating the virus. Both mechanisms may be active during dense phase carbon dioxide treatment (DPCD) which has also been shown to effectively inactivate viruses (31). The warm atmospheric pressure CO_2_ gas during DPCD is suggested to have high viral inactivation effect by penetrating the virus capsid due to the high density of CO_2_ with a high interfacial area (α) produced by the continuous CO_2_-moist contact surface area (29). Following this; CO_2_ can bind inside the capsid proteins through acid/base interactions (35), producing the high virus inactivation rates (18). Also, when compared with other gases (Air, O_2_, N_2_, and Argon), CO_2_ gas has the highest inactivated viruses and bacteria rates in different NaCl solutions, even at ambient temperatures and normal atmospheric pressure (18). Recently, Edwards et al. demonstrate the effectiveness of aerosol administration of nasal saline comprising calcium and sodium salts diminishes exhaled particles and acts as a new natural defense against airborne pathogens in the human airways (36). Moreover, Zare and his teamwork report that spraying micron-sized water droplets can act as an effective disinfectant by causing inactivation of over 98% of the bacteria. They propose that the combined action of reactive oxygen species present in micron-size water droplets (but not in bulk water) along with the droplet surface charge is responsible for the observed bactericidal activity (37). The efficiency of CO_2_ technology will require adjustment and control of the mechanical and dynamic behavior of moist CO_2_ bubbles and properties such as temperature, flow and density rates, pressure, electrolyte pH, bubble size and thickness, surfaces area, and duration. All of these factors contribute to the observed fast microbial death (38).

### Safe and Tolerance for Human Clinical Trials and Treatment

Carbon dioxide (CO_2_) gas is natural, inexpensive, non-toxic at low concentrations (5,000 ppm), non-flammable, and readily available in high purity from a variety of sources. When CO_2_ gas dissolves in water, it exists in chemical equilibrium with carbonic acid (pH = 4.18) which plays an essential role in the bicarbonate buffer system used to maintain acid-base homeostasis in the human body. The duration and concentration of carbon dioxide inhalation may be the key to the effective and protective role of CO_2_ gas therapy. A recent study investigated that pre-treatment by CO_2_ inhalation for 10 min, but not for 60 min, could improve lipopolysaccharide LPS-induced lung injury (39). A pre-clinical sheep model used perflubron combined with 12% CO_2_ to re-open constricted airways treatment for severe acute asthma (40). As a reference, OSHA has set a CO_2_ permissible exposure limit (PEL) of 5,000 ppm over 8 h and 30,000 ppm over 10 min. This compares favorably to CO gas at 50 ppm, NO gas at 25 ppm, and O_3_ gas at 0.10 ppm for 8 h. Humans can tolerate up to 10% CO_2_ before severe adverse effects are encountered (41) although CO_2_ tolerance decreases with age (*p* <0.0001) (42). Two clinical trials (NCT02616770 & NCT02334553) showed that perflubron carried in gas with ascending doses of carbon dioxide (4, 8, and 12% CO_2_) administered to healthy subjects was safe and effective in subjects with mild asthma (43, 44), while other ongoing clinical trials (NCT03903913) are testing the safety and tolerability the same formulation in subjects with cystic fibrosis. Moreover, CO_2_ concentrations of up to 35% have been applied in other clinical trial study used “CO_2_ inhalation challenge model” through a protected inhalation system to measure the anxiolytic and panicolytic effects of new test compounds (45, 46).

### Suppressing Cytokine Storm

Evidence is accumulating inferring that a subcategory of patients with acute COVID-19 might experience cytokine storm syndrome (47). CO_2_ gas is one of the potential treatment strategies to dampen an overactive immune system and to quell a cytokine storm (48, 49). Many researchers have reported that the presence of CO_2_ reduces the production of proinflammatory cytokines such as tumor necrosis factor-alpha (TNF-α) and interleukins 1 and 6 (IL-1 and IL-6, respectively), suggesting that the gas temporarily inhibits macrophage activity via a mechanism that could be associated with the reduction of the local or systemic pH (50–54). Carbon dioxide gas can also affect the production of pro-and anti-inflammatory cytokines in endotoxin-stimulated human whole blood cultures under hypercapnic, normocapnic, and hypocapnic conditions (55). In another study, CO_2_ was shown to differentially affect the cytokine release of macrophage subpopulations exclusively via alteration of extracellular pH. Decreasing the extracellular pH to 6.5 mimicked the effects of CO_2_ and a decrease to 5.5 suppressed IL-6 release in cell lines (53).

### Inhaled Carrier Gas Delivery System

CO_2_ gas has unique safety, chemical stability, biocompatibility, and properties as well as a higher density than oxygen, high solubility in tissue and blood and high tolerance in vascular system (56). CO_2_ itself is a respiratory stimuli, enhances mucus clearance, and seems to be a bronchodilator by general induction of smooth muscle relaxation (57). Additionally, warmed and humidified CO_2_ insufflation leads to an improved body core temperature (BCT) maintenance, a reduction of the inflammatory and cytokine responses (58, 59) and improved quality of postoperative course, compared with standard insufflation (60, 61). Also, it can reduce intraoperative hypothermia, coagulation dysfunction, early postoperative cough pain, days to first flatus and solid food intake, and the length of hospital stays (62). In recent years, CO_2_-based technologies have accordingly gained considerable interest in the pharmaceutical industry. CO_2_ bubble-generating carrier systems can be used to locally accumulate a drug at diseased tissue, reducing side effects on the healthy tissue and improving their therapeutic effectiveness (63). CO_2_ may also be used as an enhancer and carrier gas for delivery of effective medical agents into a surgical wound (64) or respiratory diseases such as severe acute asthma and cystic fibrosis (40, 43, 44).

### Clinical Usage and Medical Purposes

Medical carbon dioxide has been used as a pure gas or in specialized mixtures with other gases in anesthesia, as an insufflation gas for minimally invasive surgery (65), and in carboxytherapy (66). It can be used in the expansion of blood vessels to increase carbon dioxide level after rapid breathing, and to stimulate breathing after a period of non-breathing (67). Transdermal carbon dioxide gas therapy is widespread and uses carbon dioxide gas at high humidity, to increase tissue blood flow. Tissue oxygenation generates new blood vessels, and well-oxygenated tissues improve the effectiveness of antibiotic therapy. This is complemented by the antioxidant effect of CO_2_ itself, which reduces oxidative stress in open surgery (68), and improves wound healing (69).

### Benefits of Hypercapnic Therapy

Hypercapnic therapy (elevated CO_2_ levels) has beneficial effects on the physiology of the respiratory, cardiovascular, and nervous system. In human critical care, hypercapnic acidosis (HCA) is frequently acceptable and improves innate immune function, resistance to infection, and protects and improves lung functions in patients with advanced lung disease. However, all these benefits require careful consideration of when and for how long hypercapnia will be applied. Hypercapnic acidosis, but not buffered hypercapnia, was reported to reduce the severity of sepsis-induced lung injury (70). Recent studies suggest that HCA is protective in the earlier phases of bacterial pneumonia-induced sepsis, just as HCA is protective in preclinical models of early and prolonged systemic sepsis (71). Also, CO_2_ gas in therapeutic hypercapnia and other forms of acidosis techniques is an excellent antioxidant and anti-inflammatory agent (72). Hypercapnic acidosis was associated with benefits on lung and distant organs in several disease models, apart from the reduction of ventilation parameters such as ventilator-induced lung injury (73), acute respiratory distress syndrome (ARDS) (74), ischemia-reperfusion injury (75) and sepsis (76), therapeutic hypercapnia through inspired carbon dioxide attenuated lung injury, as measured by gas exchange, reduced cytokine release, lung oedema formation, and histological lung injury. Hypercapnic acidosis improves ventilation-perfusion matching that also improves gas exchange (77), prevents oedema formation (78), clears the alveolar fluid in pulmonary oedema (79), maintains the integrity of the blood-brain-barrier and reduces neurologic deficits after trauma (80). HCA also reduces the oxidative stress that contributes to pathologic thick mucus gel formation in the lung (81, 82). It is hoped that hypercapnia therapy may offer real benefits, but well-planned and executed clinical studies will be required.

### Recent COVID-19 Contradictory Studies

The partial pressure of CO_2_ in the atmosphere varies between 0.03 and 0.06% (83) but forms a high proportion (12.5–13.5%) with water vapor (1.3%) of the mainstream cigarette smoke (84). Recent studies have discovered the unusually low prevalence of current smoking was observed among hospitalized COVID-19 patients compared to the expected prevalence based on smoking prevalence in China. This preliminary analysis does not support the argument that current smoking is a risk factor for hospitalization for COVID-19, and might even suggest a protective role (85). Other cross-sectional studies in both COVID-19 out- and in-patients strongly suggests that daily smokers have a very much lower probability of developing symptomatic or severe SARS-CoV-2 infection as compared to the general population (86, 87). However, on the other hand, researchers at Baylor College of Medicine, the University of South Carolina and other institutions have identified tobacco smoking as a potential risk factor for infection of the COVID-19 virus, due to increasing the expression of ACE2, the receptor of SARS-CoV-2, in the lungs (88, 89). These two contradictory studies support our hypothesis of moist warm CO_2_ gas resulted from cigarettes smoking could kill the SARS-CoV-2 viruses inside the infected lungs of smoker patients, and that leads to decreasing the infected COVID-19 patient from the smoker, not the nicotinic or other outcomes of mainstream cigarette.

## Testing the Hypothesis (a): Preclinical Study and Inactivation Mechanisms

Herein, we recommend preclinical studies to optimize the relation between disinfection efficacy and toxicity level of warm humidified CO_2_ gas while considering other related parameters to discover the possible mechanism of action of disinfection by CO_2_ gas. The temperature inside healthy lungs is around 37°C, the pH is between 7.38 and 7.42, and the relative humidity ranges from 30 to 70%. It is essential to keep humidity stable as too high humidity provides optimal conditions for microbial growth, and low humidity and dry air can dry mucous membranes and make them more susceptible to infection (90). The SARS-CoV-2 virus is highly stable at 4°C, but it is very sensitive to heat. It is remarkably stable in a wide range of pH values (pH 3–10) at room temperature (22°C) (91, 92). However, the stability of SARS-CoV-2 under different environmental conditions of temperature, pressure, relative humidity, and pH with biological tissue and barriers require further investigation.

## Testing the Hypothesis (b): Clinical Evaluation and Implications

Whilst the properties and clinical applications of CO_2_ have been known for many decades; parameters must be systematically studied before it can be used in a new clinical setting.

### (I) Healthy, Non-symptomatic, Mild, and Moderate Care Levels

**Figure 2:**
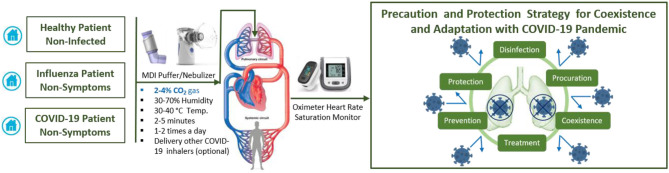
Suggested protocol for early and daily inhaling CO_2_ gas (2–4%) itself or composite with other COVID-19 inhaler medications could help in precaution and protection strategy to coexistence and adaptation with COVID-19 pandemic.

**Figure 3:**
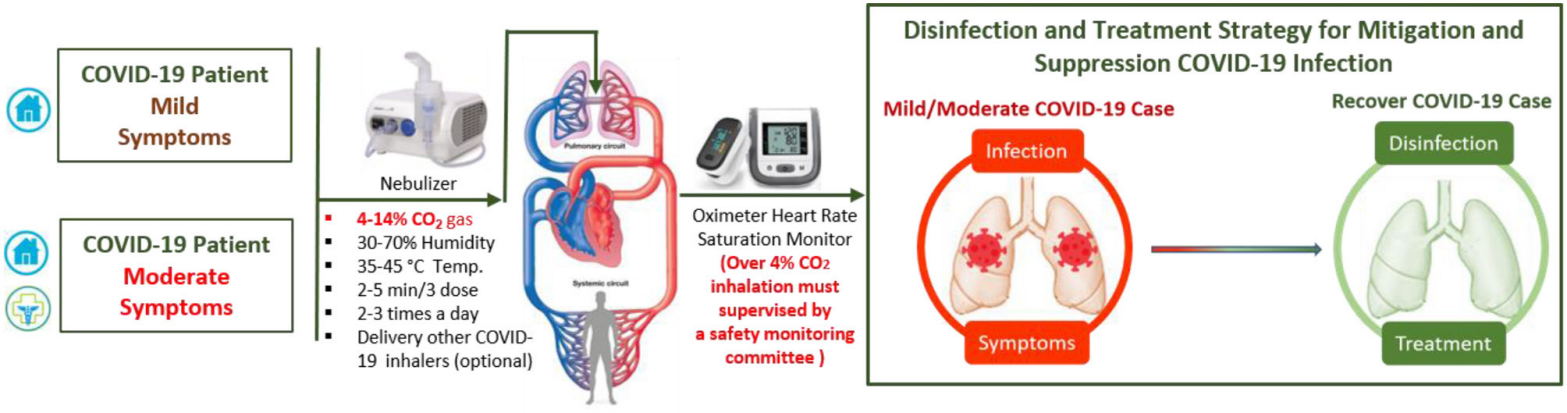
Suggested protocol for early inhaling CO_2_ gas (4–14%) itself or composite with other COVID-19 inhaler medications could save lives by disinfection and improve mild and moderate COVID-19 patient treatment.

**Figure 4:**
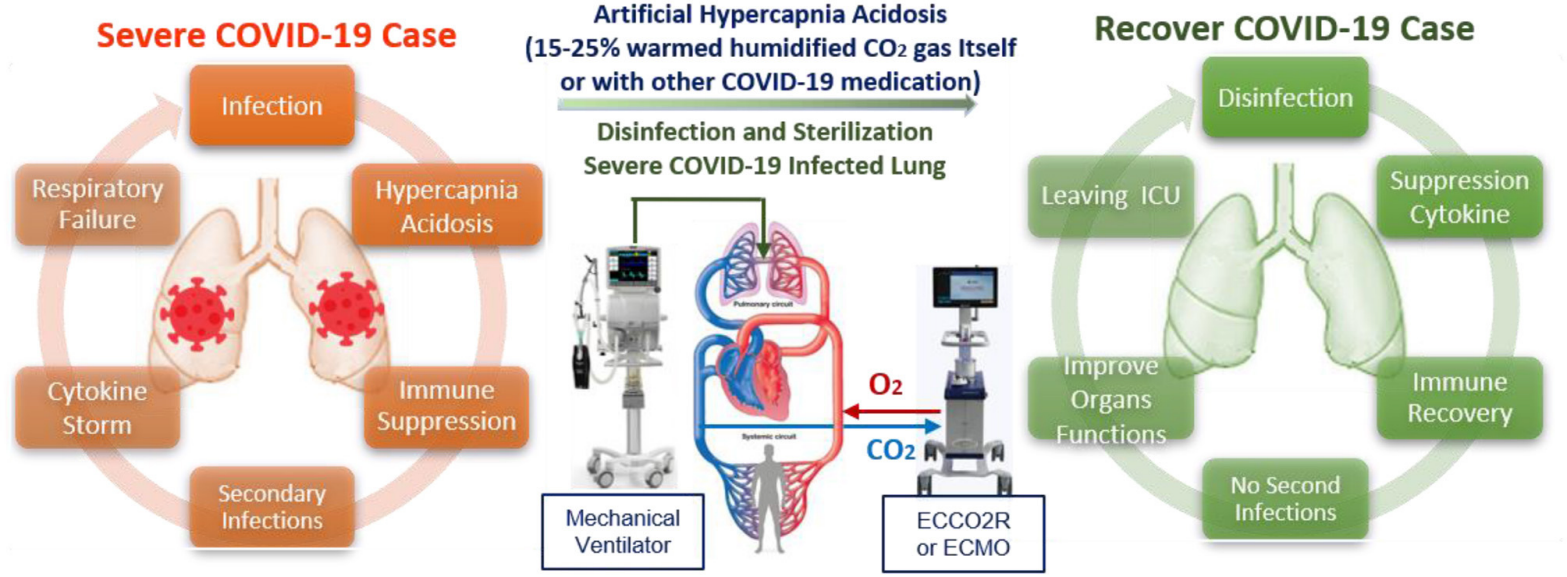
Suggested supporting treatment protocol depending on time and duration of limited and convenient artificial hypercapnia acidosis (15–25%) that could help in saving more lives of severe COVID-19 patients.

Optimizing the balance between disinfection efficacy and toxicity of humidified warmed CO_2_ gas considering other parameters (temperature, relative humidity, pressure flow and density rates, electrolyte pH, bubble size and thickness, surfaces area, and duration) will be key. Different regimes will be needed to protect healthy and non-symptomatic patients and improve the condition of those suffering mild and moderate COVD-19 symptoms. Multiple-ascending dose studies in which subjects with mild to moderate COVID-19 will be enrolled [CO_2_ max 14%, tolerance decreases with age (*p* <0.0001)] (42). The suggested study could consist of a screening period, a run-in, dosing and evaluation periods, and a follow-up period. The dosing and evaluation period of the study could divide into three connected components. *First, a dose-escalation study*—This segment of the treatment period is designed to assess the safety and tolerability of escalating doses of medical CO_2_ gas (2–4%) in a healthy volunteer (Figure 2), and (4, 8, 12, and 14%) in those with mild-moderate COVID-19 symptoms (Figure 3). *Second, a daily dosing study*- This segment of the treatment period is designed to assess the short term (5 days) safety and tolerability of 1–2 times daily administrations of a fixed dose of medical CO_2_ gas in healthy volunteers, and 2–3 times daily administration of a fixed dose of medical CO_2_ gas in patients with mild-moderate COVID-19. *Third, a drug delivery study*- This segment of the treatment period is designed to assess the safety, efficacy, enhancing, and tolerability of humidified warmed CO_2_ gas (2–14%) composed with other inhaled medication such as an antiviral (Remdesivir or IFN-β SNG001), short-acting bronchodilator, antibiotic, anti-inflammation. The recommended clinical trial study may well-include placebo-control, humidified warmed CO_2_ gas (2–14%), and humidified warmed CO_2_ gas (2–14%) composed with other inhaled medication. Administration can be achieved through using simple comprised cartridge MDI puffer, portable nebulizer, or circularize II high-efficiency aerosol drug delivery system nebulizer in a negative pressure environment. Direct air/oxygen inhalation for a few minutes can be used to recover patients to baseline carbon dioxide levels. A safety monitoring committee must also review the results from each cohort before deciding continuation of the study at the next prescribed dose level, based on consideration of the clinical significance of safety and tolerability parameters.

### (II) Severe Care Level

The damage mechanisms of SARS-CoV-2 are still unclear, with severe COVID-19 cases are complicated by high mortality rates due to compromised immune function and a high probability of antibiotic-resistant secondary infections. Most severe COVID-19 cases are associated with respiratory failure, with many already suffering from internal high hypercapnia acidosis (with humidity levels near 100%) that disrupt not only cardiac and neurological functions but also immune system function by suppressing both innate and adaptive immune responses to viral and bacterial proliferation and infection (54, 93–96). This dysfunction of the immune system with increasing SARS-CoV-2 infection can lead to an overreaction of the immune system (cytokine storm), during which white blood cells are misdirected to attack and inflame even healthy tissue, leading to failure of the lungs, heart, liver, intestines, kidneys, and genitals (Multiple Organ Dysfunction Syndrome, MODS). This may, in turn, lead to the lungs shutting down (Acute Respiratory Distress Syndrome, ARDS), which makes absorption of oxygen difficult. Most deaths due to COVID-19 are due to respiratory failure. To save the lives of severing COVID-19 patients, we must first stop the causes of SARS-CoV-2 infection and preventing secondary infections. However, due to the absence of a specific COVID-19 antiviral treatment, most severe COVID-19 patients be admitted to the intensive care unit to fight the symptoms, aiming to lower the mortality rate through intensive monitoring and supportive organ function treatments by anti-cytokine medications with artificial blood purification system machines (97). Herein, we cautiously suggest that external artificial hypercapnia acidosis (warmed humidified CO_2_ 15–25%) could be applied to disinfect and stabilize the lungs of SARS-CoV-2 infected patients and prevent secondary infections (Figure 4). However, it should only be considered for severely affected patients if they are already is connected to life support and artificial blood purification through mechanical means, and a controlled gas mixture consisting of 25% CO_2_ and 75% O_2_ is delivered through a protected inhalation system while monitoring a wide range of physiological parameters, and administering supportive organ function treatments.

## Conclusion and Expecting Outcomes

There is an urgent global need for a universal vaccine to cover all SARS-CoV-2 mutant strains to stop the threat of an inevitable second wave of coronavirus. Currently, there are hundreds of clinical trials, but not yet any approved antiviral drugs specific for the treatment of COVID-19. The physical, biological, and medical properties of CO_2_ gas suggest that humified warmed CO_2_ gas possesses multiple bioactivities and offer a new concept to SARS-CoV-2 viral disinfection and COVID-19 treatment. This inexpensive and broadly applicable therapy could lead to a massive reduction in the global number of infected, especially when used as a carrier for delivery of other inhaled drugs and creates new possibilities for mitigation and suppression of any COVID-19 second wave, or indeed any new future respiratory viral pandemic. In the future, more bioactive properties of CO_2_ could be identified, and their mechanisms of action investigated. We believe well-designed clinical trials of CO_2_ and its various bioactive properties are warranted to examine its efficacy against these diseases in human beings. It is hoped that this hypothesis will serve as a stimulus for further investigation into this issue.

## Data Availability Statement

The original contributions presented in the study are included in the article/supplementary material, further inquiries can be directed to the corresponding author/s.

## Author Contributions

All authors contributed to the article and approved the submitted version. AE-B: conceptualization, methodology, writing—original draft, and writing—review and editing. EB: writing—review and editing. MG and KH: conceptualization and writing—review and editing.

## Conflict of Interest

The authors declare that the research was conducted in the absence of any commercial or financial relationships that could be construed as a potential conflict of interest.

## References

[1] Oxley TJ Mocco J Majidi S Kellner CP Shoirah H Singh IP Large-vessel stroke as a presenting feature of covid-19 in the young. N Engl J Med. (2020) 382:e60 10.1056/NEJMc2009787 32343504 PMC7207073

[2] Sungnak W Huang N Bécavin C Berg M Queen R Litvinukova M. SARS-CoV-2 entry factors are highly expressed in nasal epithelial cells together with innate immune genes. Nat Med. (2020) 26:681–7. 10.1038/s41591-020-0868-6 32327758 PMC8637938

[3] Liu Y Ning Z Chen Y Guo M Liu Y Gali NK. Aerodynamic analysis of SARS-CoV-2 in two Wuhan hospitals. Nature. (2020) 582:557–60. 10.1038/s41586-020-2271-3 32340022

[4] Tang X Wu C Li X Song Y Yao X Wu X On the origin and continuing evolution of SARS-CoV-2. Natl Sci Rev. (2020) 3:nwaa036 10.1093/nsr/nwaa036 PMC7107875 34676127

[5] Shi Y Wang Y Shao C Huang J Gan J Huang X. COVID-19 infection: the perspectives on immune responses. Cell Death Differ. (2020) 27:1451–4. 10.1038/s41418-020-0530-3 32205856 PMC7091918

[6] Chen N Zhou M Dong X Qu J Gong F Han Y. Epidemiological and clinical characteristics of 99 cases of 2019 novel coronavirus pneumonia in Wuhan, China: a descriptive study. Lancet. (2020) 395:507–13. 10.1016/S0140-6736(20)30211-7 32007143 PMC7135076

[7] Callaway E. The race for coronavirus vaccines: a graphical guide. Nature. (2020) 580:576–7. 10.1038/d41586-020-01221-y 32346146

[8] Ma Z Pu F Zhang X Yan Y Zhao L Zhang A. Carbon monoxide and biliverdin suppress bovine viral diarrhoea virus replication. J Gen Virol. (2017) 98:2982–92. 10.1099/jgv.0.000955 29087274

[9] Fredenburgh LE Kraft BD Hess DR Harris RS Wolf MA Suliman HB. Effects of inhaled CO administration on acute lung injury in baboons with pneumococcal pneumonia. Am J Physiol Lung Cell Mol Physiol. (2015) 309:L834–46. 10.1152/ajplung.00240.2015 26320156 PMC4609940

[10] Fredenburgh LE Perrella MA Barragan-Bradford D Hess DR Peters E Welty-Wolf KE. A phase I trial of low-dose inhaled carbon monoxide in sepsis-induced ARDS. JCI Insight. (2018) 3:e124039. 10.1172/jci.insight.124039 30518685 PMC6328240

[11] Barrese E Gioffrè A Scarpelli M Turbante D Trovato R Iavicoli S Indoor pollution in work office: VOCs, formaldehyde and ozone by printer. Occupat Dis Environ Med. (2014) 3:49–55. 10.4236/odem.2014.23006

[12] Dubuis ME Dumont-Leblond N Laliberté C Veillette M Turgeon N. Ozone efficacy for the control of airborne viruses: bacteriophage and norovirus models. PLoS ONE. (2020) 15:e0231164. 10.1371/journal.pone.0231164 32275685 PMC7147755

[13] Rowen RJ Robins H A plausible “Penny” costing effective treatment for corona virus ozone therapy. J Infect Dis Epidemiol. (2020) 6:113 10.23937/2474-3658/1510113

[14] Matthay MA Aldrich JM Gotts JE. Treatment for severe acute respiratory distress syndrome from COVID-19. Lancet Respir Med. (2020) 8:433–4. 10.1016/S2213-2600(20)30127-2 32203709 PMC7118607

[15] Martel J Ko YF Young JD Ojcius DM. Could nasal nitric oxide help to mitigate the severity of COVID-19? Microb Infect. (2020) 22:168–71. 10.1016/j.micinf.2020.05.002 32387333 PMC7200356

[16] Ichinose F Roberts JD Jr Zapol WM. Inhaled nitric oxide: a selective pulmonary vasodilator: current uses and therapeutic potential. Circulation. (2004) 109:3106–11. 10.1161/01.CIR.0000134595.80170.62 15226227

[17] Martirosyan V Hovnanyan K Ayrapetyan S Carbon dioxide as a microbial toxicity enhancer of some antibacterial agents: a new potential water purification tool. ISRN Biophys. (2012) 2012:906761 10.5402/2012/906761

[18] Garrido Sanchis A Pashley R Ninham B Virus and bacteria inactivation by CO_2_ bubbles in solution. NPJ Clean Water. (2019) 2:5 10.1038/s41545-018-0027-5

[19] Fages Jacques Patrick Frayssinet and Gilbert Bonel “Uses for a current of supercritical carbon dioxide as an antiviral agent.” U.S. Patent No. 5,723,012. 3 Mar. 1998. Washington, DC: U.S. Patent and Trademark Office.

[20] Fages J Frayssinet P Bonel G Antiviral treatment of collagenous material for use as prostheses and grafts-by treating with supercritical carbon di:oxide, hydrogen peroxide, sodium hydroxide and ethanol, preventing viral contamination from e.g. Hepatitis C. Patent EP748632-A1; FR2735372-A1; US5723012-A; EP748632-B1; DE69619893-E; ES2174035-T3. Bioland Sarl; Depuy Bioland (1997).

[21] Qiu Y Lin G Zhang M Chen Q Method for removing the activity of coronavirus by using supercritical fluid. Patent CN1721526-A; CN1318581-C. Nanwei Ind Co Ltd (2006).

[22] Balestrini JL Liu A Gard AL Huie J Blatt KMS Schwan J Sterilization of lung matrices by supercritical carbon dioxide. Tissue Eng C Methods. (2016) 22:260–9. 10.1089/ten.tec.2015.0449 PMC4782026 26697757

[23] Persson M Svenarud P Flock JI Van Der Linden J Carbon dioxide inhibits the growth rate of *Staphylococcus aureus* at body temperature. Surg Endosc Other Interv Tech. (2005) 19:91–4. 10.1007/s00464-003-9334-z 15529188

[24] Rizzo AN Dudek SM. Endothelial glycocalyx repair: building a wall to protect the lung during sepsis. Am J Respir Cell Mol Biol. (2017) 56:687–88. 10.1165/rcmb.2017-0065ED 28569597 PMC5516297

[25] Curry FE. The molecular structure of the endothelial glycocalyx layer (EGL) and surface layers (ESL) modulation of transvascular exchange. Adv Exp Med Biol. (2018) 1097:29–49. 10.1007/978-3-319-96445-4_2 30315538

[26] Sieve I Münster-Kühnel AK Hilfiker-Kleiner D. Regulation and function of endothelial glycocalyx layer in vascular diseases. Vascul Pharmacol. (2018) 100:26–33. 10.1016/j.vph.2017.09.002 28919014

[27] Reines BP Ninham BW. Structure and function of the endothelial surface layer: unraveling the nanoarchitecture of biological surfaces. Q Rev Biophys. (2019) 52:e13. 10.1017/S0033583519000118 31771669

[28] Isenschmid A Marison I Von Stockar U. The influence of pressure and temperature of compressed CO_2_ on the survival of yeast cells. J Biotechnol. (1995) 39:229–37. 10.1016/0168-1656(95)00018-L 7766403

[29] Garrido A Pashley R Ninham B Water sterilisation using different hot gases in a bubble column reactor. J Environ Chem Eng. (2018) 6:2651–9. 10.1016/j.jece.2018.04.004

[30] Lin HM Yang Z Chen LF Inactivation of saccharomyces cerevisiae by supercritical and subcritical carbon dioxide. Biotechnol Progr. (1992) 8:458–61. 10.1021/bp00017a013

[31] Balaban MO Ferrentino G. Dense Phase Carbon Dioxide: Food and Pharmaceutical Applications. John Wiley and Sons. Ames, IA (2012). 10.1002/9781118243350

[32] Becker Z A comparison between the action of carbonic acid and other acids upon the living cell. Protoplasma. (1936) 25:161–75. 10.1007/BF01839067

[33] Debs-Louka E Louka N Abraham G Chabot V Allaf K. Effect of compressed carbon dioxide on microbial cell viability. Appl Environ Microbiol. (1999) 65:626–31. 10.1128/AEM.65.2.626-631.1999 9925592 PMC91071

[34] Cheng X Imai T Teeka J Hirose M Higuchi T Sekine M. Inactivation of bacteriophages by high levels of dissolved CO_2_. Environ Technol. (2013) 34:539–44. 10.1080/09593330.2012.704403 23530369

[35] Cundari TR Wilson AK Drummond ML Gonzalez HE Jorgensen KR Payne S. CO_2_-formatics: how do proteins bind carbon dioxide? J Chem Inform Model. (2009) 49:2111–15. 10.1021/ci9002377 19705826

[36] Edwards D Hickey A Batycky R Griel L Lipp M Dehaan W A new natural defense against airborne pathogens. QRB Discov. (2020) 1:e5 10.1017/qrd.2020.9 PMC7453358 34192261

[37] Dulay MT Lee JK Mody AC Narasimhan R Monack DM Zare RN Spraying small water droplets acts as a bacteriocide. QRB Discov. (2020) 1:e3 10.1017/qrd.2020.2 PMC10392691 37528962

[38] Spilimbergo S Bertucco A. Non-thermal bacterial inactivation with dense CO_2_. Biotechnol Bioeng. (2003) 84:627–38. 10.1002/bit.10783 14595775

[39] Tang SE Wu SY Chu SJ Tzeng YS Peng CK Lan CC. Pre-treatment with ten-minute carbon dioxide inhalation prevents lipopolysaccharide-induced lung injury in mice via down-regulation of toll-like receptor 4 expression. Int J Mol Sci. (2019) 20:6293. 10.3390/ijms20246293 31847115 PMC6940754

[40] E Mays T Choudhury P Leigh R Koumoundouros E Velden J Shrestha G Nebulized perflubron and carbon dioxide rapidly dilate constricted airways in an ovine model of allergic asthma. Respir Res. (2014) 15:98 10.1186/s12931-014-0098-x 25355286 PMC4172894

[41] Zaba C Marcinkowski JT Wojtyła A Tezyk A Tobolski J Zaba Z. Acute collective gas poisoning at work in a manure storage tank. Ann Agric Environ Med. (2011) 18:448–51. 22216829

[42] Gill M Natoli MJ Vacchiano C MacLeod DB Ikeda K Qin M. Effects of elevated oxygen and carbon dioxide partial pressures on respiratory function and cognitive performance. J Appl Physiol. (2014) 117:406–12. 10.1152/japplphysiol.00995.2013 24947022

[43] Green FHY Leigh R Fadayomi M Lalli G Chiu A Shrestha G A phase I, placebo-controlled, randomized, double-blind, single ascending dose-ranging study to evaluate the safety and tolerability of a novel biophysical bronchodilator (S-1226) administered by nebulization in healthy volunteers. Antimicrob Agents Chemother. (2016) 61:e00279–17. 10.1186/s13063-016-1489-8 PMC4964056 27464582

[44] Swystun V Green FHY Dennis JH Rampakakis E Lalli G Fadayomi M. A phase IIa proof-of-concept, placebo-controlled, randomized, double-blind, crossover, single-dose clinical trial of a new class of bronchodilator for acute asthma. Trials. (2018) 19:321. 10.1186/s13063-018-2720-6 29914544 PMC6006836

[45] Leibold NK van den Hove DLA Viechtbauer W Buchanan GF Goossens L Lange I. CO_2_ exposure as translational cross-species experimental model for panic. Transl Psychiatry. (2016) 6:e885. 10.1038/tp.2016.162 27598969 PMC5048202

[46] Vickers K Jafarpour S Mofidi A Rafat B Woznica Linett A. The 35% carbon dioxide test in stress and panic research: overview of effects and integration of findings. Clin Psychol Rev. (2012) 32:153–64. 10.1016/j.cpr.2011.12.004 22322014

[47] Yuen KS Ye ZW Fung SY Chan CP Jin DY. SARS-CoV-2 and COVID-19: the most important research questions. Cell Biosci. (2020) 10:40. 10.1186/s13578-020-00404-4 32190290 PMC7074995

[48] Roberto Rodrigues Bicalho P Magna Ribeiro F Henrique Ferreira Marçal P Gomes de Alvarenga D de Sá Silva F. Does helium pneumoperitoneum reduce the hyperinflammatory response in septic animals during laparoscopy? Surg Res Pract. (2020) 2020:5738236. 10.1155/2020/5738236 32232117 PMC7091538

[49] Lu Z Casalino-Matsuda SM Nair A Buchbinder A Budinger GRS Sporn PHS. A role for heat shock factor 1 in hypercapnia-induced inhibition of inflammatory cytokine expression. FASEB J. (2018) 32:3614–22. 10.1096/fj.201701164R 29405096 PMC5998969

[50] West MA Baker J Bellingham J. Kinetics of decreased LPS-stimulated cytokine release by macrophages exposed to CO_2_. J Surg Res. (1996) 63:269–74. 10.1006/jsre.1996.0259 8661209

[51] Jacobi CA Ordemann J Halle E Volk HD Müller JM. Impact of laparoscopy with carbon dioxide versus helium on local and systemic inflammation in an animal model of peritonitis. J Laparoendosc Adv Surg Tech. (1999) 9:305–12. 10.1089/lap.1999.9.305 10414552

[52] Matsumoto T Tsuboi S Dolgor B Bandoh T Yoshida T Kitano S. The effect of gases in the intraperitoneal space on cytokine response and bacterial translocation in a rat model. Surg Endosc. (2001) 15:80–84. 10.1007/s004640000293 11178769

[53] Kos M Kuebler J Jesch N Vieten G Bax N van der Zee D. Carbon dioxide differentially affects the cytokine release of macrophage subpopulations exclusively via alteration of extracellular Ph. Surg Endosc Other Int Tech. (2006) 20:570–6. 10.1007/s00464-004-2175-6 16437285

[54] Hanly EJ Aurora AR Fuentes JM Shih SP Marohn MR De Maio A Hypercapnia and acidosis in sepsis – anesthesiology. J Gastrointest Surg. (2005) 9:1245–52. 10.1097/ALN.0b013e3181ca361f 16332480

[55] Kimura D Totapally BR Raszynski A Ramachandran C Torbati D. The effects of CO_2_ on cytokine concentrations in endotoxin-stimulated human whole blood. Crit Care Med. (2008) 36:2823–7. 10.1097/CCM.0b013e318186f556 18766096

[56] Martens S Neumann K Sodemann C Deschka H Wimmer-Greinecker G Moritz A. Carbon dioxide field flooding reduces neurologic impairment after open heart surgery. Ann Thoracic Surg. (2008) 85:543–7. 10.1016/j.athoracsur.2007.08.047 18222261

[57] Van den Elshout F Van Herwaarden C Folgering H. Effects of hypercapnia and hypocapnia on respiratory resistance in normal and asthmatic subjects. Thorax. (1991) 46:28–32. 10.1136/thx.46.1.28 1908137 PMC1020910

[58] Oderda M Cerutti E Gontero P Manetta T Mengozzi G Meyer N. Standard insufflation during RARP. Minerva Anestesiol. (2018) 84:1228. 10.23736/S0375-9393.18.12695-2 29633813

[59] Oderda M Cerutti E Gontero P Manetta T Mengozzi G Meyer N. The impact of warmed and humidified CO_2_ insufflation during robotic radical prostatectomy: results of a randomized controlled trial. Urol J. (2019) 86:130–40. 10.1177/0391560319834837 30868938

[60] Schlotterbeck H Schaeffer R Dow WA Diemunsch P. Cold nebulization used to prevent heat loss during laparoscopic surgery: an experimental study in pigs. Surg Endosc. (2008) 22:2616–20. 10.1007/s00464-008-9841-z 18347861

[61] Noll E Schaeffer R Joshi G Diemunsch S Koessler S Diemunsch P. Heat loss during carbon dioxide insufflation: comparison of a nebulization based humidification device with a humidification and heating system. Surg Endosc. (2012) 26:3622–5. 10.1007/s00464-012-2385-2 22722768

[62] Jiang R Sun Y Wang H Liang M Xie X. Effect of different carbon dioxide (CO_2_) insufflation for laparoscopic colorectal surgery in elderly patients: a randomized controlled trial. Medicine. (2019) 98:e17520. 10.1097/MD.0000000000017520 31593122 PMC6799792

[63] Lin YJ Huang CC Wan WL Chiang CH Chang Y Sung HW. Recent advances in CO_2_ bubble-generating carrier systems for localized controlled release. Biomaterials. (2017) 133:154–64. 10.1016/j.biomaterials.2017.04.018 28437626

[64] Persson M van der Linden J. The potential use of carbon dioxide as a carrier gas for drug delivery into open wounds. Med Hypotheses. (2009) 72:121–4. 10.1016/j.mehy.2008.08.026 18990501

[65] Winkler JL Jeronimo J Singleton J Janmohamed A Santos C. Performance of cryotherapy devices using nitrous oxide and carbon dioxide. Int J Gynecol Obstetr. (2010) 111:73–77. 10.1016/j.ijgo.2010.04.032 20580000

[66] Verrier N Fournier C Fournel T. 3D tracking the brownian motion of colloidal particles using digital holographic microscopy and joint reconstruction. Appl Opt. (2015) 54:4996–5002. 10.1364/AO.54.004996 26192657

[67] Yu T Cheng Y Wang X Tu B Cheng N Gong J. Gases for establishing pneumoperitoneum during laparoscopic abdominal surgery. Cochrane Database Syst Rev. (2017) 6:CD009569. 10.1002/14651858.CD009569.pub3 28635028 PMC6481852

[68] Tsuchiya M Sato EF Inoue M Asada A. CO_2_ field flooding may also reduce oxidative stress in open surgery. Anesth Anal. (2009) 109:683–4. 10.1213/ane.0b013e3181a909be 19608848

[69] Brandi C Grimaldi L Nisi G Brafa A Campa A Calabrò M. The role of carbon dioxide therapy in the treatment of chronic wounds. In Vivo. (2010) 24:223–6. 20363999

[70] Higgins BD Costello J Contreras M Hassett P O' Toole D Laffey JG. Differential effects of buffered hypercapnia versus hypercapnic acidosis on shock and lung injury induced by systemic sepsis. Anesthesiology. (2009) 111:1317–26. 10.1097/ALN.0b013e3181ba3c11 19934878

[71] Curley G Contreras M Nichol A Higgins B Laffey J. Hypercapnia and acidosis in sepsis. Anesthesiology. (2010) 112:462–72. 10.1097/ALN.0b013e3181ca361f 20068449

[72] Gates KL Howell HA Nair A Vohwinkel CU Welch LC Beitel GJ. Hypercapnia impairs lung neutrophil function and increases mortality in murine pseudomonas pneumonia. Am J Respir Cell Mol Biol. (2013) 49:821–8. 10.1165/rcmb.2012-0487OC 23777386 PMC3931098

[73] Peltekova V Engelberts D Otulakowski G Uematsu S Post M Kavanagh BP. Hypercapnic acidosis in ventilator-induced lung injury. Intensive Care Med. (2010) 36:869–78. 10.1007/s00134-010-1787-7 20213072

[74] Hummler HD Banke K Wolfson MR Buonocore G Ebsen M Bernhard W The effects of lung protective ventilation or hypercapnic acidosis on gas exchange and lung injury in surfactant deficient rabbits. PLoS ONE. (2016) 11:e0147807 10.1371/journal.pone.0147807 26840779 PMC4739580

[75] Laffey JG Jankov RP Engelberts D Tanswell AK Post M Lindsay T. Effects of therapeutic hypercapnia on mesenteric ischemia–reperfusion injury. Am J Respir Crit Care Med. (2003) 168:1383–90. 10.1164/rccm.2108078 14644926

[76] O'Toole D Hassett P Contreras M Higgins BD McKeown STW McAuley DF. Hypercapnic acidosis attenuates pulmonary epithelial wound repair by an NF-κB dependent mechanism. Thorax. (2009) 64:976–82. 10.1136/thx.2008.110304 19617214

[77] Sinclair SE Kregenow DA Starr I Schimmel C Lamm WJE Hlastala MP. Therapeutic hypercapnia and ventilation-perfusion matching in acute lung injury: low minute ventilation vs inspired CO_2_. Chest. (2006) 130:85–92. 10.1378/chest.130.1.85 16840387

[78] Ketabchi F Egemnazarov B Schermuly RT Ghofrani HA Seeger W Grimminger F. Effects of hypercapnia with and without acidosis on hypoxic pulmonary vasoconstriction. Am J Physiol Lung Cell Mol Physiol. (2009) 297:L977–83. 10.1152/ajplung.00074.2009 19717554

[79] Briva A Lecuona E Sznajder JI. Permissive and non-permissive hypercapnia: mechanisms of action and consequences of high carbon dioxide levels. Arch Bronconeumol. (2010) 46:378–82. 10.1016/S1579-2129(10)70088-4 20303638 PMC3858013

[80] Yang WC Wang Q Chi LT Wang YZ Cao HL Li WZ. Therapeutic hypercapnia reduces blood–brain barrier damage possibly via protein kinase Cε in rats with lateral fluid percussion injury. J Neuroinflammation. (2019) 16:36. 10.1186/s12974-019-1427-2 30760300 PMC6375143

[81] Yuan S Hollinger M Lachowicz-Scroggins ME Kerr SC Dunican EM Daniel BM. Oxidation increases mucin polymer cross-links to stiffen airway mucus gels. Sci Transl Med. (2015) 7:276ra27. 10.1126/scitranslmed.3010525 25717100 PMC4403633

[82] Fahy JV Dickey BF. Airway mucus function and dysfunction. N Engl J Med. (2010) 363:2233–47. 10.1056/NEJMra0910061 21121836 PMC4048736

[83] Keeling CD Whorf TP Atmospheric CO_2_ Records from Sites in the Scripps Institution of Oceanography (SIO) Air Sampling Network (1985–2007). (2004). 10.3334/CDIAC/ATG.NDP001

[84] Guais A Brand G Jacquot L Karrer M Dukan S Grévillot G. Toxicity of carbon dioxide: a review. Chem Res Toxicol. (2011) 24:2061–70. 10.1021/tx200220r 21732636

[85] Farsalinos K Barbouni A Niaura R Smoking, vaping and hospitalization for COVID-19. Qeios. [Preprint] (2020) 10.32388/Z69O8A.5

[86] Miyara M Tubach F Amoura Z Low incidence of daily active tobacco smoking in patients with symptomatic COVID-19. Qeios [Preprint]. (2020) 10.32388/WPP19W

[87] Changeux JP Amoura Z Rey FA Miyara M. A nicotinic hypothesis for Covid-19 with preventive and therapeutic implications. Qeios. (2020) 343:33–39. 10.32388/FXGQSB.2 32720486

[88] Cai G Bossé Y Xiao F Kheradmand F Amos CI. Tobacco smoking increases the lung gene expression of ACE2, the receptor of SARS-CoV-2. Am J Respir Crit Care Med. (2020) 201:1557–9. 10.1164/rccm.202003-0693LE 32329629 PMC7301735

[89] Patanavanich R Glantz SA. Smoking is associated with COVID-19 progression: a meta-analysis. Nicotine Tob Res. (2020) 22:1653–6. 10.1093/ntr/ntaa082 32399563 PMC7239135

[90] Lepeule J Litonjua AA Gasparrini A Koutrakis P Sparrow D Vokonas PS. Lung function association with outdoor temperature and relative humidity and its interaction with air pollution in the elderly. Environ Res. (2018) 165:110–17. 10.1016/j.envres.2018.03.039 29684737 PMC5999568

[91] Chin AWH Chu JTS Perera MRA Hui KPY Yen HL Chan MCW. Stability of SARS-CoV-2 in different environmental conditions. Lancet Microbe. (2020) 1:e10. 10.1016/S2666-5247(20)30003-3 32835322 PMC7214863

[92] van Doremalen N Bushmaker T Morris DH Holbrook MG Gamble A Williamson BN Aerosol and surface stability of SARS-CoV-2 as compared with SARS-CoV-1. N Engl J Med. (2020) 382:1564–7. 10.1056/NEJMc2004973 32182409 PMC7121658

[93] Coakley R Taggart C Greene C McElvaney N O'Neill S. Ambient pCO_2_ modulates intracellular pH, intracellular oxidant generation, and interleukin-8 secretion in human neutrophils. J Leukocyte Biol. (2002) 71:603–10. 10.1189/jlb.71.4.603 11927646

[94] Vadász I Hubmayr RD Nin N Sporn PH Sznajder JI. Hypercapnia: a nonpermissive environment for the lung. Am J Respir Cell Mol Biol. (2012) 46:417–21. 10.1165/rcmb.2011-0395PS 22246860 PMC3359943

[95] Pugin J Dunn-Siegrist I Dufour J Tissieres P Charles PE Comte R. Cyclic stretch of human lung cells induces an acidification and promotes bacterial growth. Am J Respir Cell Mol Biol. (2008) 38:362–70. 10.1165/rcmb.2007-0114OC 17921360

[96] Helenius IT Krupinski T Turnbull DW Gruenbaum Y Silverman N Johnson EA. Elevated CO_2_ suppresses specific drosophila innate immune responses and resistance to bacterial infection. Proc Natl Acad Sci USA. (2009) 106:18710–15. 10.1073/pnas.0905925106 19846771 PMC2773965

[97] Xie P Ma W Tang H Liu D. Severe COVID-19: a review of recent progress with a look toward the future. Front Public Health. (2020) 8:189. 10.3389/fpubh.2020.00189 32574292 PMC7237759

[98] El-Betany A Behiry E Gumbleton M Harding K Humidified warmed CO_2_ treatment therapy strategies can save lives with mitigation and suppression of SARS-CoV-2 infection: an evidence review. OSF Preprints [Preprint]. (2020) 10.31219/osf.io/7tj2g PMC7793941 33425942

